# A Neck-Thyroid Phantom with Small Sizes of Thyroid Remnants for Postsurgical I-123 and I-131 SPECT/CT Imaging

**DOI:** 10.3390/life13040961

**Published:** 2023-04-06

**Authors:** Konstantinos Michael, Anastasia Hadjiconstanti, Antonis Lontos, George Demosthenous, Savvas Frangos, Yiannis Parpottas

**Affiliations:** 1 Department of Mechanical Engineering, Frederick University, Nicosia 1036, Cyprus; 2 Department of Medical Physics, Bank of Cyprus Oncology Center, Nicosia 2006, Cyprus; 3 Frederick Research Center, Nicosia 1036, Cyprus; 4 Department of Nuclear Medicine, Bank of Cyprus Oncology Center, Nicosia 2006, Cyprus

**Keywords:** thyroid-neck phantom, thyroid remnants, postsurgical diagnostic thyroid imaging, nuclear imaging

## Abstract

Post-surgical I-123 and I-131 SPECT/CT imaging can provide information on the presence and sizes of thyroid remnants and/or metastasis for an accurate re-staging of disease to apply an individualized radioiodine therapy. The purpose of this study was to develop and validate a neck–thyroid phantom with small sizes of thyroid remnants to be utilized for the optimization of post-surgical SPECT/CT imaging. 3D printing and molding techniques were used to develop the hollow human-shaped and -sized phantom which enclosed the trachea, esophagus, cervical spine, clavicle, and multiple detachable sections with different sizes of thyroid remnant in clinically relevant positions. CT images were acquired to evaluate the morphology of the phantom and the sizes of remnants. Triple-energy window scattered and attenuation corrected SPECT images were acquired for this phantom and for a modified RS-542 commercial solid neck–thyroid phantom. The response and sensitivity of the SPECT modality for different administered I-123 and I-131 activities within the equal-size remnants of both phantoms were calculated. When we compared the phantoms, using the same radiopharmaceutical and similar activities, we found that the measured sensitivities were comparable. In all cases, the I-123 counting rate was higher than the I-131 one. This phantom with capabilities to insert different small sizes of remnants and simulate different background-to-remnants activity ratios can be utilized to evaluate postsurgical thyroid SPECT/CT imaging procedures.

## 1. Introduction

Differentiated thyroid cancer (DTC) treatment typically involves surgical removal of the whole or the largest part of the thyroid gland and a subsequent radioiodine therapy (RAIT). RAIT involves the administration of iodide (I-131) for selective irradiation of thyroid remnants, microscopic DTC or non-resectable or incompletely resectable DTC [1].

Activities administered as part of RAIT are generally empirically determined and fixed by a given institution based on disease characteristics and patient age. The administration of fixed therapeutic doses implies that these procedures do not apply personalized medicine, which means that some patients may inevitably be administered larger or even lower doses than the dose that would most likely result in the greatest benefit. Moreover, recent DTC management guidelines [2] propose “selective” use of radioiodine ablation for patients considered as being at “low-risk” or even “intermediate risk” of DTC recurrence or mortality. In a previous retrospective study [3], it was observed that patients with putatively “low-intermediate risk” DTC frequently had disease characteristics denoting high or uncertain risk, suggesting that “selective” radioiodine ablation in such patients may not be applicable.

Post-surgical nuclear medicine imaging can provide further information on the presence of thyroid remnants and/or metastasis for an accurate (re-)staging of each patient’s disease following surgery, thus ensuring a more informative decision for radioiodine ablation. This imaging can be performed with simple pinhole scintigraphy, planar scintigraphy or single photon emission tomography (SPECT). For SPECT imaging, Tc-99m, I-131 or I-123 labelled radiopharmaceuticals can be employed, with I-123 being favorable because, unlike Tc-99m, it has the same physical characteristics as I-131, which is used for ablation, while avoiding the “stunning” effect [4] and lower image quality, which is observed when the latter is used [5,6,7]. These scans may be utilized for visual evaluation by physicians, estimation of remnant volume [8,9,10] and pre-ablation dosimetry [11] to further aid treatment decisions. 

An anthropomorphic neck–thyroid phantom with different small sizes of thyroid remnants in clinically relevant positions is important for optimizing the postsurgical thyroid-remnant SPECT/CT imaging procedures such as protocols, radiopharmaceuticals and activities, scatter correction algorithms, as well as volume and dose calculation algorithms. 

Previous studies [8,9,10] developed simple water containers with hollow cylindrical or spherical inserts down to 5 mL to simulate thyroid remnants. They utilized these phantoms to validate algorithms using Tc-99m SPECT images. Mortelmans et al. [8] also used different background-to-remnant activity ratios. 

Physical phantoms with thyroid glands have been developed and evaluated in previous studies [12,13,14,15]. In particular, solid cylindrical phantoms, with and without other structures such as the trachea, esophagus and cylindrical cervical spine, and a detachable section of the thyroid gland have been developed and evaluated using Tc-99m or I-131 SPECT images [14,15]. Alqahtani et al. [15] developed a hollow head–neck phantom which includes the trachea, cylindrical cervical spine, a non-detachable thyroid gland and different sizes of sentinel lymph nodes. This was evaluated using Tc-99m SPECT-CT. 

The purpose of this paper is, firstly, to present the development of a dedicated phantom for postsurgical thyroid-remnant SPECT/CT imaging, secondly, to validate its anatomy using CT images, and, finally, to evaluate its performance in I-131 and I-123 SPECT/CT imaging. 

## 2. Materials and Methods

### 2.1. Designs

The human-sized and -shaped neck–thyroid phantom is separated in three main parts: the hollow sealed cavity of the neck phantom, the detachable sections with thyroid remnants, and the structures within the hollow cavity of the neck phantom including the cervical spine, the clavicle, the trachea, and esophagus. 

**Figure 1:**
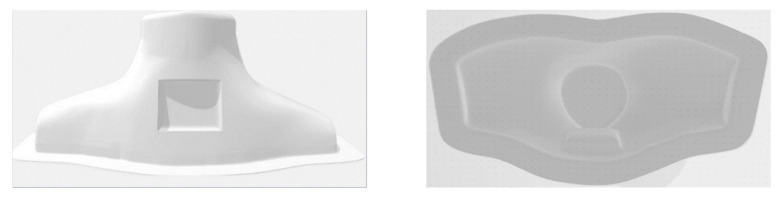
(**Left**) Front and (**right**) bottom views of the design for the neck phantom. The front view also shows the outer hollow cavity which is filled with the detachable thyroid-remnants section.

A SolidWorks software package (Version 28) [16] was utilized for the designs. For the size of the neck phantom, publicly available DICOM images [17] were utilized, and then modified according to the shape of the commercial RS-542 solid–neck phantom [18]. Figure 1 shows the design of the neck phantom. The wall thickness was 2 mm. Two openings of 20- and 3 mm in diameter were drawn at the top part of the neck for filling the phantom with water and injecting the diluted radiopharmaceutical, respectively. The corresponding caps and threads were also designed for these openings. 

**Figure 2:**
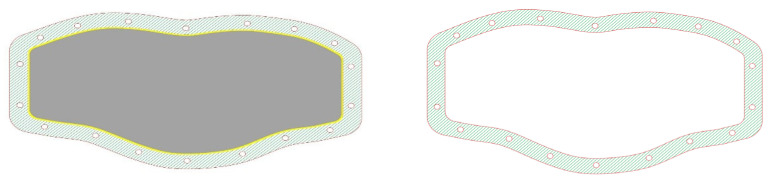
The designs of (**left**) the base of the phantom and (**right**) the ring. The peripheral openings for the screws and the peripheral groove (yellow) of the O-ring are also shown.

A 10 mm-thick base was designed to cover the bottom part of the phantom. Eighteen small openings were drawn around the periphery of the base to be tightened to the neck phantom with screws. They were centered at 11 mm from the outer periphery of the base. A groove was also drawn around the periphery of the base to place a 5 mm-in-diameter O-ring and optimize the seal between the base and the neck phantom. It was centered at 17 mm from the outer periphery of the base. In addition, two 10-mm-thick rings of the same size and shape as the bottom flat part of the phantom were also designed. Eighteen small openings were drawn on the rings to match the corresponding openings on the base. The rings are positioned above and below the bottom flat part of the phantom, together with the base and the O-ring, to provide the optimal seal. Figure 2 shows the designs of the base and the ring.

**Figure 3:**
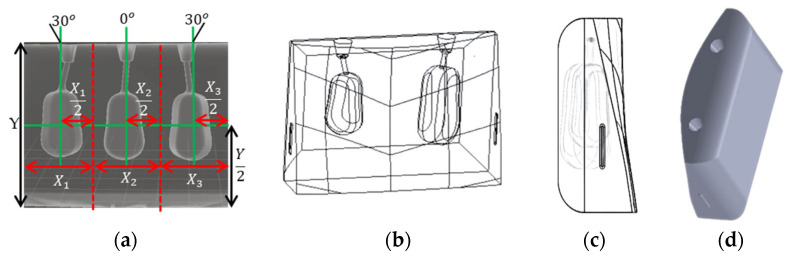
Designs of sections with (**a**) three thyroid remnants of equal size (1.5 mL) where their geometrical position within the section is presented, and (**b**) two thyroid remnants of 1.5 and 3 mL centered in areas X_1_ and X_3_, respectively; (**c**) side view of a section showing the clip that is used to attach the section to the phantom; (**d**) top view of a section showing the two openings that are used to inject a radiopharmaceutical within the corresponding two remnants.

**Figure 4:**
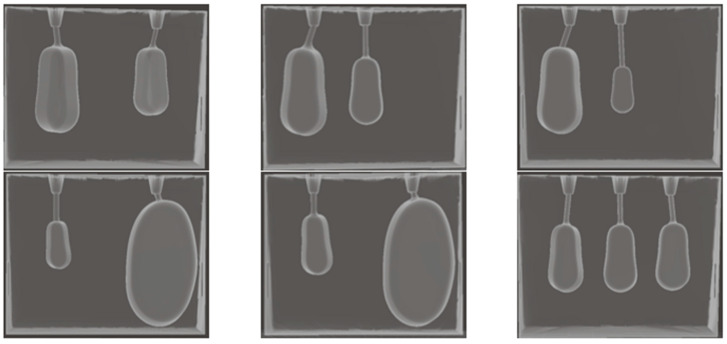
The designed sections with thyroid remnants of: (**top-left** and **top-middle**) 3 and 1.5 mL at different positions, (**top-right**) 3 and 0.5 mL, (**bottom-left**) 0.5 and 10 mL, (**bottom-middle**) 1 and 10 mL, and (**bottom-right**) 1.5 mL each.

Six sections (7 × 5 × 1.5 cm^3^) with two or three hollow cavities (thyroid remnants) of different sizes (0.5–10 mL), at clinically relevant positions after thyroidectomy, were designed as shown in Figure 3 and Figure 4. Each section can be easily added and removed from the front outer hollow cavity of the phantom, as shown in Figure 1. The section width was separated into three equal-sized areas as shown in Figure 3a. A remnant was horizontally and vertically centered in an area. The left and right remnants were rotated by 30° to fit into a section. Tiny openings were drawn at the top of a section to inject diluted radiopharmaceuticals into the hollow cavities of the remnants.

**Figure 5:**
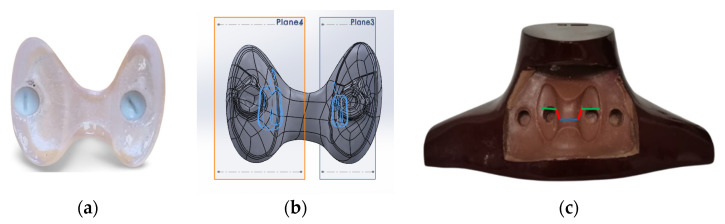
(**a**) Back view of the thyroid gland of the RS-542 neck–thyroid phantom; (**b**) back view of the design of the modified RS-542 thyroid gland with 3- and 1.5-mL remnants (blue); (**c**) front view of the RS542 neck–thyroid phantom where the three most common clinically relevant areas with remnants after thyroidectomy are presented with different colors.

In addition, the thyroid gland of the RS-542 phantom was (re-)designed to include the hollow cavities of the remnants as shown in Figure 5b. In total, six thyroid glands were designed, including remnants of the same size and in the same positions as those of the custom-made phantom, shown in Figure 4. In this case, the width of the gland was separated into three areas as denoted by different colors in Figure 5c. These modifications were performed in order to compare the counting rate from the commercial RS-542 phantom and the custom-made phantom in postsurgical thyroid SPECT/CT imaging, when activity was administered only within the remnants.

**Figure 6:**
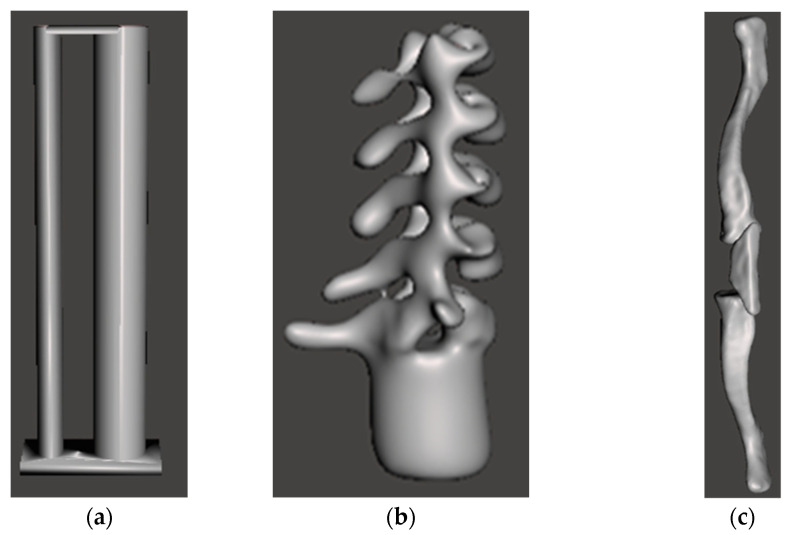
Designs of the hollow cavities of (**a**) esophagus (left part) and trachea (right part) structure; (**b**) cervical spine; and (**c**) clavicle. The dimensions are listed in the second table.

The trachea, esophagus, cervical spine, and clavicle were designed to be anatomically positioned, and glued, on the base of the phantom. Both the trachea and the esophagus were designed as 2-mm-thick hollow and air-filled elliptical cylinders with closed ends. They were also connected at both ends to ensure stability, as shown in Figure 6. The lateral and anterior-posterior axes of trachea [19] were equal to 1.3 cm and 1 cm, respectively, while the corresponding ones for the esophagus [20] were 1 cm and 0.3 cm, respectively. 

For the design of the cervical spine and clavicle, volumetric data from CT images of the Cancer Genome Atlas Thyroid Cancer Collection [17,21] were converted to 3D-printed models (STL format), as described by Bücking et al. [22] using a 3D Slicer software package (Version 4.8.1) [23]. Next, the 3D models were modified according to the size of the neck phantom, and according to the corresponding cervical and clavicle of the RS-542 phantom, using the SolidWorks software [16]. The designs of the spine and clavicle are shown in Figure 6.

### 2.2. Developments

**Figure 7:**
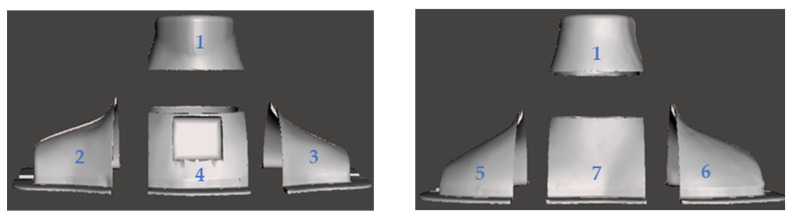
(**Left**) Front and (**right**) back views of the phantom design, separated into seven pieces for 3D-printing.

The neck phantom was developed utilizing 3D-printing (Form 2, FormLabs [24]) and molding techniques. The design was printed using a transparent resin (Clear Resin V4) with a density of 1.09 g/cm^3^. Seven separate pieces were printed because of the limited volume of the 3D-printer and then glued together to form the neck phantom, as shown in Figure 7. Two 3D-printed phantoms were used to construct a mold assembly, and hence to be able to develop the neck phantom in one piece within a short time period. Thus, the time-consuming printing procedure, as well as possible leaks of water and radiopharmaceuticals from the glued joints, can be avoided. 

**Figure 8:**
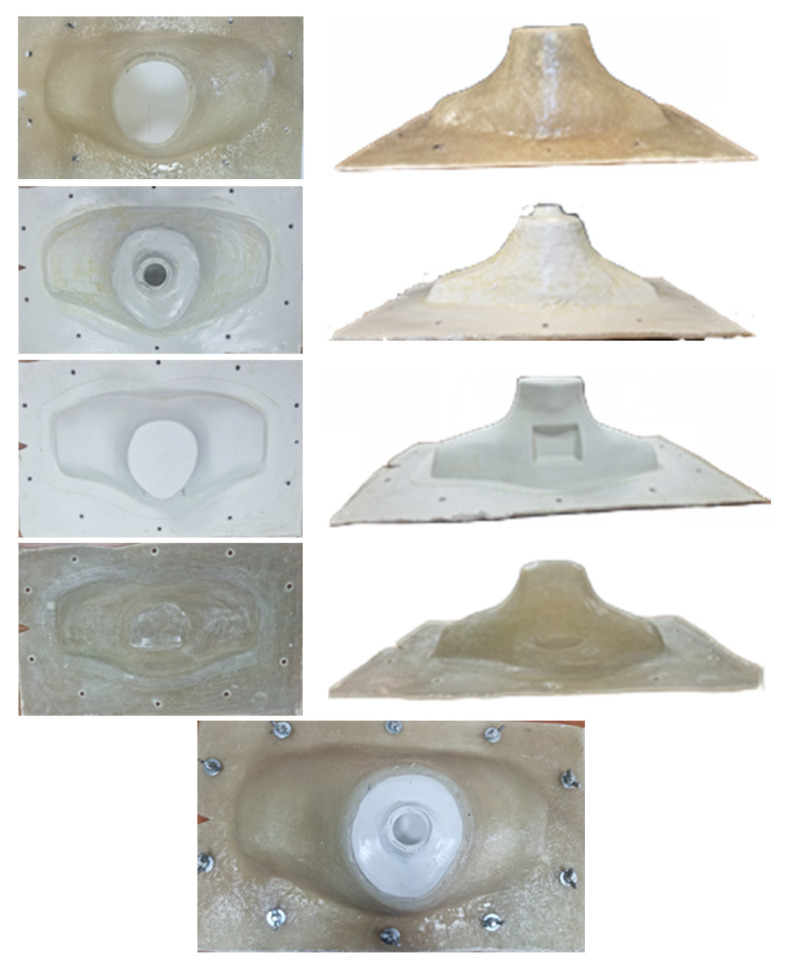
(**Left column**) Top and (**right column**) front views of the four successive pieces of the mold assembly: each piece is placed within each other, where the top piece is shown in the first row and the bottom piece in the fourth row. The pieces are made of fiberglass (first and fourth rows) and silicone (second and third rows). (Last row) The assembled and tightened phantom assembly.

The mold assembly consists of two fiberglass and two silicone pieces. Figure 8 shows the consecutive pieces in order as placed on top of the other to form the mold. When all pieces of the mold are tightened together with screws, a 2-mm-thick cavity is formed between the two silicone (second and third) pieces to pour within a liquid resin [25] and produce the neck phantom. The resin density was close to 1 g/cm^3^ to simulate human tissue. The resin could be dried within a short time. The above-mentioned materials were chosen because the loose silicone pieces could be easily disassembled from the produced neck phantom and because the hard plexiglass (first and fourth) pieces provided a stable shape for the mold. 

To manufacture the fiberglass pieces, firstly, all areas of the 3D-printed phantoms were perfectly sanded before applying a mold-release wax. Next, fiberglass plies were laid on the inner and outer areas of the 3D-printed phantom, and brushed with laminating resin, to form the first and fourth pieces of the mold. Multiple layers of fiberglass plies and laminating resin were used to produce the fiberglass pieces. 

In order to manufacture the second and third pieces (silicone) of the mold, liquid silicone was poured within two structures which were placed as follows: (a) the first piece (fiberglass) on top of the outer part of the 3D-printed phantom to form the second piece, and (b) the inner part of the 3D-printed phantom on top of the fourth piece (fiberglass) to form the third piece. During the molding procedure, multiple 5-mm-thick wooden spacers were temporarily placed between the corresponding two structures—at the bottom periphery of the structures—to form a specific thickness for the silicone pieces. 

**Figure 9:**
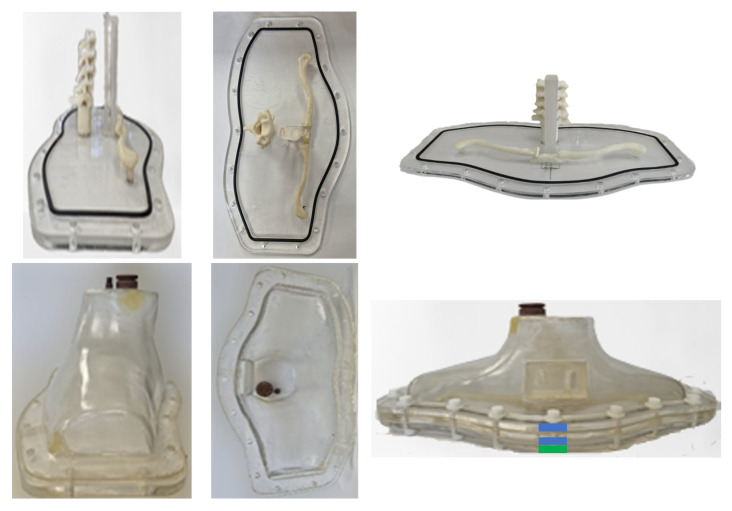
(**Top-row**) Different views of the phantom base on which the trachea, esophagus, cervical spine, clavicle, and O-ring are attached, and (**bottom-row**) different views of the phantom together with top caps made of solid water material. (**Bottom-right**) The sealed, and tightened with screws, neck–thyroid phantom with a detachable section of 1.5- and 3-mL thyroid remnants; the vertical thickness of the base is denoted with a green color and the corresponding thicknesses of the two rings are denoted with a blue color.

Figure 9 shows the neck phantom produced using the above mold assembly. A drill was used for the two openings at the top part of the neck phantom. The corresponding threads and gaps for these openings were manufactured using solid water material. The threads were glued to the specific openings as shown in Figure 9. 

A laser cutting-engraver machine and plexiglass material were utilized to form the base of the phantom according to the design. Eighteen openings for the screws and the groove for the O-ring around the periphery of the base, as well as the two rings, were also manufactured using the same machine. Different views of the base as well as the sealed neck phantom with the rings and the base are shown in Figure 9.

The sections with thyroid remnants for the custom-made neck–thyroid phantom and the thyroid glands with thyroid remnants for the RS-542 neck–thyroid phantom were 3D-printed as one piece. In Figure 9, a section with thyroid remnants of 1.5 and 3 mL is attached to the neck phantom. The diluted radiopharmaceutical could be injected via a tiny opening at the top of the hollow cavity of a remnant, as shown in Figure 3, and it could be sealed using a hot glue gun. 

The hollow cavities of the trachea, esophagus, cervical spine, and clavicle were also 3D-printed. Tiny openings were added at various peripheral points of the cervical spine and the clavicle designs so as to be able to inject uniformly within the 3D-printed models the resin material [26] of 1.74 g/cm^3^ in density. 

### 2.3. CT and SPECT/CT Acquisitions

A Siemens Somatom Sensation Open CT scanner (Siemens Healthcare, Erlangen, Germany) of the Bank of Cyprus Oncology Center was utilized to characterize the neck–thyroid phantom with thyroid remnants. High-resolution CT images were acquired at 120 kV and 200 mAs, with a slice thickness of 0.6 mm, and reconstructed using multiplanar reformation (MPR). These images were used to evaluate the anatomy and calculate the volume of the various cavities within the phantom. The volume of remnants was calculated by measuring the cross-sectional area of each axial slice using ImageJ image analysis software (Version 1.53) [27], and then by adding them, as described by Brenner et al. [28]. The calculated volume cavities using CT images were compared with the corresponding volumes from the 3D designs. 

The dual-head GE Infinia Hawkeye 4 SPECT/4-slice-CT hybrid scanner (GE Healthcare, Milwaukee, WI, USA) of the Bank of Cyprus Oncology Center was utilized to acquire images and evaluate the performance of the postsurgical thyroid SPECT imaging procedure using the developed phantom with diluted I-131 and I-123. First, the uniformity of the uptake in the remnants of the custom-made phantom was investigated by studying the count profile and the full width at half maximum (FWHM) when administering I-131 or I-123 in the remnants. Second, the response (R = counts/activity) and sensitivity (S = R/time) of the SPECT/CT modality to the 1.5- and 3-mL remnants of the custom-made and commercial phantoms were calculated and compared when administering I-131 or I-123. 

All acquisitions were performed following the clinical protocols. For I-131, two high energy general purpose (HEGP) collimators in 180° (H mode) orientation were used. SPECT data were acquired in 60 projections, 35 s per projection, over 180° of rotation thus covering an angular range of 360°, using a matrix size of 128 × 128. The data were reconstructed using the ordered-subset expectation-maximization (OSEM) algorithm with two iterations and ten subsets. A Butterworth filter (cut-off: 0.48, power: 10) was applied on the reconstructed images. SPECT data were scattered corrected using the Triple-Energy Window (TEW) method, as described by Hadjiconstanti et al. [29]. For this purpose, three energy windows were set during the I-131 acquisitions: (a) a ±10% main window around the 364 keV photopeak of I-131 (327.6–400.4 keV), (b) a 306.9 keV ± 0.81% left sub-window (304.4–309.4 keV), and (c) a 421.1 keV ± 0.59% right sub-window (418.6–423.6 keV). CT images with 5 mm-slice thickness were acquired using a matrix size of 512 × 512, and were used for attenuation correction. 

I-123 acquisitions were performed using two low energy high resolution (LEHR) collimators. For the TEW scatter correction, the following energy windows were set: (a) a ±10% energy window centered over the 159 keV photopeak of I-123 (143.1–174.9 keV), (b) a 130 keV ± 1.95% left sub-window (127.5–132.5 keV), and (c) a 179.9 keV ± 1.55% right sub-window (177.5–182.5 keV). The rest of the acquisitions and reconstruction parameters were similar to the corresponding I-131 acquisitions. 

## 3. Results

**Figure 10:**
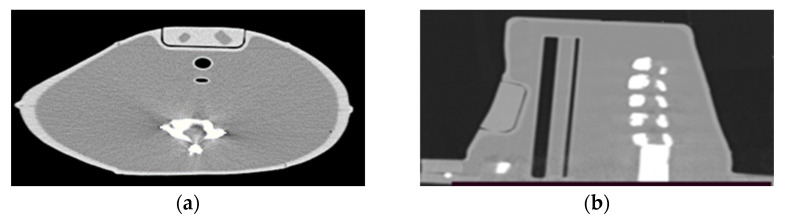

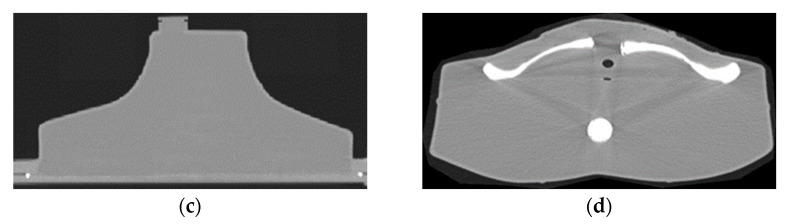
(**a**) Axial CT slice through the center of the 1.5- and 3-mL thyroid remnants; (**b**) sagittal CT slice through the center of the spine; (**c**) coronal slice through the center of the phantom (between esophagus and cervical spine); and (**d**) axial CT slice through the bottom part of the phantom showing a top view of the clavicle. The phantom was filled with water.

Figure 10 shows an axial CT slice through the center of the 1.5- and 3-mL thyroid remnants, a sagittal CT slice through the center of the spine, a coronal slice through the center of the phantom (between the esophagus and cervical spine) and an axial CT slice through the bottom part of the phantom showing a top view of the clavicle. It should be noted that the hollow cavities of the neck phantom and the remnants were filled with water. It can be observed that the structures within the phantom were anatomically positioned according to a sectional anatomy atlas [30], and their densities were uniform. In addition, the X-ray contrast between the various structures was satisfactory according to the sectional anatomy atlas [30] for distinguishing the structures. Only minor streaking artifacts can be observed close to the bones. 

In Table 1, the remnant volumes, as defined by the SolidWorks [16], were compared with the corresponding measured volumes by using CT images and the ImageJ software [27]. Multiple sections with thyroid remnants of different sizes were imaged, their volumes were measured, and the average measured volumes were reported. The average % error between the defined and measured volumes of remnants was 1.5%, indicating that the volume of a specific remnant can be accurately calculated from CT images. This is important in postsurgical thyroid SPECT/CT imaging because accurate volume calculation can lead to an accurate calculation of the ablative dose for a remnant.

Similarly, Table 2 presents the defined dimensions for the other structures of the phantom and the corresponding measured dimensions, as described above. The average % error between the defined and measured dimensions was 2.8%, indicating that the human-sized dimensions of the structures can be easily identified from CT images.

**Figure 11:**
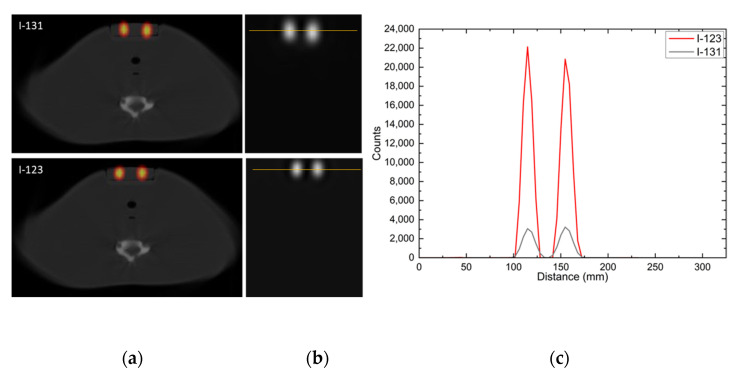
(**a**) Axial fused TEW I-131 and I-123 SPECT/CT slices through the middle of the 1.5- (left part of the slice) and 3-mL (right part of the slice) remnants; (**b**) the corresponding line profiles; and (**c**) the distributed counts within the remnants according to the line profiles. I-131 and I-123 administered activities are given within the text.

Figure 11 presents the axial fused TEW SPECT/CT slices through the middle of the remnants as well as the corresponding line profiles. The administered activity for the I-131 acquisitions was 4.7 MBq and 5 MBq for the 1.5- and 3-mL remnants, respectively. For I-123 acquisitions, the administered activity was 5.7 MBq for both remnants. In addition, plots for the corresponding FWHM are also shown. The FWHM for the 1.5-mL remnant was 15.4 mm and for the 3-mL remnant was 16 mm—when I-131 was administered—while the corresponding FWHM for the 1.5-mL remnant was 13.3 mm and for the 3-mL remnant was 14.1 mm, when I-123 was administered. The measured I-123 counts within each remnant were about 5.5 times higher than the I-131 counts within the corresponding remnants. Both the resolution and counting rate of I-123 were higher than the corresponding I-131 ones. 

**Figure 12:**
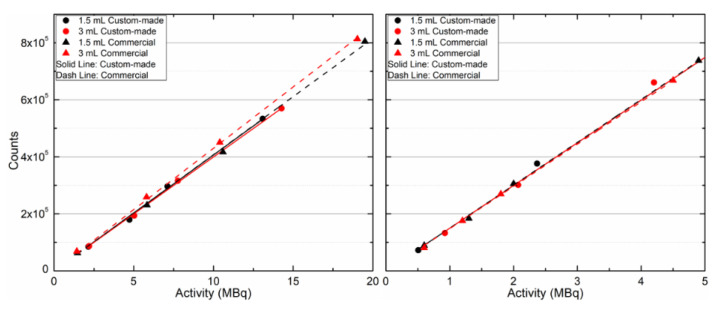
Counts as a function of the administered activity for (**left**) I-131 and (**right**) I-123 of the 1.5- and 3-mL thyroid remnants of the custom-made and commercial phantoms. A linear fit was applied to each dataset.

Figure 12 presents the counts as a function of the administered I-131 and I-123 activity within the 1.5- and 3-mL remnants of the custom-made and commercial phantoms for the TEW SPECT/CT images. I-131 activities ranged from 2.2 to 19.5 MBq while the I-123 activities ranged from 0.5 to 5.7 MBq. Four data points were obtained for each remnant size, radiopharmaceutical, and phantom. The count rate of I-123 was higher than I-131. Each dataset was linearly fitted. A correlation was observed in all cases. Consequently, the SPECT/CT modality responded linearly to the increase in the administered activity for the sizes of the examined remnants, for both radiopharmaceuticals, and for both phantoms. 

Table 3 shows the calculated sensitivity values, and the average sensitivity per dataset, S in C/MBq∙s, where C is the total counts in the remnant. It can be observed that the average sensitivity for the same radiopharmaceutical, and for the two different sizes of remnants and the two different phantoms are in agreement within the standard deviation. Thus, the diluted radiopharmaceuticals were uniform within the cavity of the remnants in both phantoms. 

**Figure 13:**
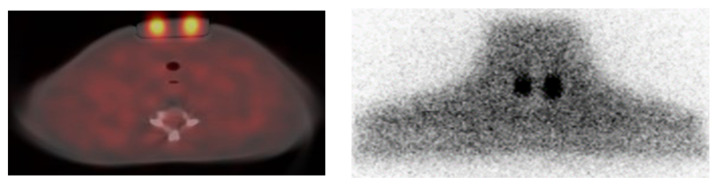
(**Left**) Fused SPECT/CT slice through the centre of the remnants and (**right**) planar anterior-posterior (AP) image of the custom-made phantom with an administered I-131 activities of 0.37 MBq/mL within the remnants and a background-to-remnant activity ratio of 5%.

Figure 13 presents a fused SPECT/CT slice and a planar/static anterior-posterior (AP) acquisition of the custom-made phantom with an administered I-131 activity of 0.37 MBq/mL within the remnants, and a background-to-remnant activity ratio of 5%. The uniformity of the background activity was assessed by taking several ROIs in different slices of the background region, using the ImageJ software, to calculate the coefficient of variation $COV=SD/mean_{ROI}\times 100\%$ [31]. It can be observed that the background activity was uniform. The calculated COV from the SPECT image was 7.8% whereas the corresponding COV from the static AP image was 5%.

**Figure 14:**
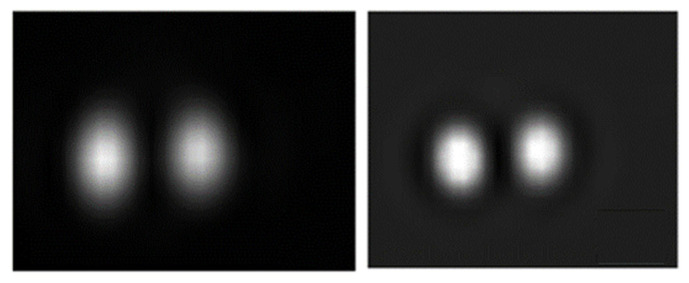
The TEW scatter corrected (**left**) I-131 and (**right**) I-123 SPECT/CT images using the custom-made phantom with 1.5- and 3-mL thyroid remnants. In both acquisitions, the administered I-131 and I-123 activities within the remnants were 13 MBq.

Figure 14 shows the I-131 and I-123 TEW scatter corrected SPECT/CT images of the custom-made phantom with 1.5- and 3-mL remnants. In both acquisitions, the administered I-131 and I-123 activities within the remnants were 13 MBq. An experienced nuclear medicine physician visually evaluated the images. Less spillover of activity and better uniformity were observed on the I-123 than the I-131 images. The physician was more confident in deciding the volume of remnants from I-123 than I-131 images. 

## 4. Discussion

Empirical methods to determine the ablative dose or administration of fixed doses may result in larger or even lower doses than the desirable dose with the greatest benefit. Postsurgical thyroid SPECT/CT imaging is important to accurately determine the volume of remnants, and consequently, to accurately calculate the ablative dose for remnants using dedicated algorithms.

Not only algorithms for volume calculation of remnants and calculation of the ablative dose, but also, dedicated phantoms are necessary to validate such algorithms, and to optimize the postsurgical thyroid nuclear medicine procedures.

Such a neck–thyroid phantom with the capability of easily inserting different small sizes of remnants, at different clinically relevant positions, was developed and presented. Diluted radiopharmaceuticals can be injected within the remnants and the neck cavity to simulate different background-to-remnant activity ratios. It also includes the other main structures within the neck cavity such as the trachea, esophagus, cervical spine and clavicle. 3D-printing and molding techniques were utilized for the development of the neck phantom. The presented custom-made phantom, using the abovementioned designs, can be manufactured in a short period of time using relatively inexpensive materials. The materials were chosen to be close to the densities of human tissues, and to avoid undesirable scattering in CT and SPECT/CT acquisitions.

A more detailed structure with different layers of materials could be used to better simulate the bone components. However, we chose to keep the bone structures simple, and easy to manufacture, because the specific structures did not produce any additional scattering.

In previous studies, either water containers with hollow inserts down to 5 mL were used to simulate remnants [8,9,10] or neck–thyroid phantoms with hollow thyroid glands with or without other anatomical structures [12,13,14,15] were used in Tc-99m and I-131 SPECT studies. In this study, a neck–thyroid phantom with different sizes of small (0.5–10 mL) thyroid remnants at clinically relevant positions, including all relevant anatomical structures, and able to simulate different background-to-remnant activity ratios, was developed to specifically study postsurgical thyroid-remnant SPECT/CT imaging procedures.

CT images of the custom-made phantom with 1.5- and 3-mL thyroid remnants validated the anatomical positioning of all structures within the phantom and the uniform density of the structures. Different tools from imaging software were utilized to measure the volumes and dimensions of these structures, as seen from CT images, and it was found to agree with the corresponding designed ones. Thus, CT images can be used to extract information regarding the remnant volume using related algorithms.

I-131 and I-123 SPECT/CT imaging was performed to evaluate the phantom with 1.5- and 3-mL thyroid remnants using routine clinical protocols for postsurgical thyroid SPECT/CT imaging. Both small remnants could be distinguished in the correct anatomical position. When fused SPECT/CT images of similar I-131 and I-123 administered activities were compared using the abovementioned remnants of this phantom, the resolution and counting rate with I-123 were higher than that those with I-131. It was also demonstrated that the response and sensitivity of the SPECT/CT modality were higher when using I-123 than I-131. One of the main reasons for this difference is the use of LEHR collimators for the emitted 159 keV gamma rays of I-123. In I-131 imaging, HEGP collimators are used to reduce the septal penetration due to the emitted higher energy of gamma rays (364 keV) resulting in poorer resolution, and sensitivity. The latter is in agreement with previous patient studies that compared the quality of I-123 and I-131 SPECT images [4,5,7]. In addition, the use of I-123 is preferable to I-131 for diagnostic purposes because I-123 is a pure gamma emitter with a short half-life (13.2 h) whereas I-131 is also a beta emitter—more harmful to patients—with a longer half-life (eight days).

SPECT/CT images with I-131 and I-123 of similar administered activities within the 1.5- and 3-mL remnants of the custom-made phantom and RS-542 commercial phantom, after modification of the RS-542 thyroid gland to include remnants, were compared. Both the counting rate for clinically relevant activities and sensitivity values were similar. This comparison was performed to determine whether the structures and materials of the custom-made phantom could result in any differences from those of the RS-542 phantom in I-131 and I-123 SPECT/CT imaging. However, the solid RS-542 phantom cannot be utilized in postsurgical thyroid-remnant imaging because it does not contain remnants, and additionally no background activity can be administered.

## 5. Conclusions

The role of postsurgical thyroid-remnant SPECT/CT imaging is crucial in the management of RAIT because the remnant volume and ablative dose can be accurately determined for an individualized treatment. 

In this study, a dedicated phantom for this imaging was developed and presented. It can be easily manufactured using relatively inexpensive materials, and in a short period of time because of the developed mold. Detachable sections of small thyroid remnants of different sizes (0.5–10 mL) can be positioned in clinically relevant positions, within the phantom, allowing us to easily perform acquisitions and study different remnants. The phantom also contains the trachea, esophagus, clavicle, and cervical spine, and it can simulate different background-to-remnants activity ratios. 

The phantom anatomy and the selected materials were validated using CT images. Its performance was evaluated using postsurgical thyroid-remnant SPECT/CT imaging. A direct quantitative and qualitative comparison verified the superiority of using I-123 over I-131 in this imaging procedure. 

The custom-made phantom can be further improved by including other anatomical details in the area of the neck, such as the thyroid/cricoid cartilage, and first rib, to investigate any additional scattering. In addition, the internal jugular vein can be added, where inferior deep cervical glands are located to investigate metastatic thyroid cancer to these glands. Other future work will involve the validation of the developed volume and dose calculation algorithms using this custom-made phantom in postsurgical thyroid-remnant SPECT/CT imaging. 

## Figures and Tables

**Table 1:** The defined remnant volume from the SolidWorks software and the corresponding average measured volume of remnants from a number of sections, using CT images and the ImageJ software, as well as the % error between the defined and measured volume.

Remnant Volume (mL)	No. of Sections	Measured Volume (mL)	Error (%)
0.5	3	0.49 ± 0.02	2.0
1.0	3	1.02 ± 0.03	2.0
1.5	5	1.51 ± 0.02	0.6
3.0	5	3.03 ± 0.04	1.0
10.0	3	10.17 ± 0.14	1.7
			Avg: 1.5

**Table 2:** The defined dimensions from the SolidWorks software and the measured dimensions using CT slices and the ImageJ software for the various structures of the phantom, as well as the % error between the defined and measured dimensions.

Structure	Dimension	Defined (mm)	Measured (mm)	Error (%)
Trachea	Lateral axis	13.0	13.6	4.6
	Anterior-Posterior axis	10.0	10.5	5.0
	Superior-Inferior	140.0	142.4	1.7
Esophagus	Lateral axis	10.0	10.4	4.0
	Anterior-Posterior axis	3.0	3.2	6.6
	Superior-Inferior	140.0	141.9	1.3
Spine	Superior-Inferior	87.3	87.4	0.1
	Anterior-Posterior of largest vertebrae	53.9	54.8	0.2
Clavicle (medial)	Lateral	52.0	54.0	3.8
	Anterior-Posterior (Mid)	12.7	13.0	2.4
	Superior-Inferior (Mid)	14.4	14.0	2.7
Clavicle (right)	Lateral	123.0	121.0	1.6
	Anterior-Posterior (Mid)	14.0	13.8	1.4
	Superior-Inferior (Mid)	9.0	9.4	4.4
				Avg: 2.8

**Table 3:** The sensitivity (S) and the average S per dataset in C/MBq∙s, where C is the total counts in each remnant) values as a function of the I-131 and I-123 administered activities (A in MBq) within the 1.5- and 3-mL thyroid remnants of the custom-made and the commercial phantoms.

I-131							
Custom-Made				Commercial			
1.5 mL		3 mL		1.5 mL		3 mL	
A	S	A	S	A	S	A	S
2.2	18.8	2.2	18.7	1.5	20.4	1.4	22.8
4.7	18.0	5.0	18.4	5.8	18.8	5.8	21.2
7.1	19.8	7.8	19.4	10.6	18.7	10.4	20.6
13.1	19.4	14.3	19.0	19.5	19.6	19.0	20.3
Avg. S	19.0		18.9		19.4		21.2
**I-123**							
0.5	68.2	0.9	68.2	0.6	70.8	0.6	64.2
2.2	75.6	2.0	69.2	1.3	67.3	1.2	69.6
5.2	70.3	4.2	74.9	2.0	72.8	1.8	71.3
5.7	71.3	5.7	69.7	4.9	71.7	4.5	70.7
Avg. S	71.4		70.5		70.6		69.0

## Data Availability

The data presented in this study are available upon request from the corresponding authors.
